# Role of Calmodulin-Calmodulin Kinase II, cAMP/Protein Kinase A and ERK 1/2 on *Aeromonas hydrophila*-Induced Apoptosis of Head Kidney Macrophages

**DOI:** 10.1371/journal.ppat.1004018

**Published:** 2014-04-24

**Authors:** Chaitali Banerjee, Preeti Khatri, Rajagopal Raman, Himanshi Bhatia, Malabika Datta, Shibnath Mazumder

**Affiliations:** 1 Immunobiology Laboratory, Department of Zoology, University of Delhi, Delhi, India; 2 Gut Biology Laboratory, Department of Zoology, University of Delhi, Delhi, India; 3 Institute of Genomics and Integrative Biology, CSIR, Delhi, India; University of Illinois, United States of America

## Abstract

The role of calcium (Ca^2+^) and its dependent protease calpain in *Aeromonas hydrophila*-induced head kidney macrophage (HKM) apoptosis has been reported. Here, we report the pro-apoptotic involvement of calmodulin (CaM) and calmodulin kinase II gamma (CaMKII*g*) in the process. We observed significant increase in CaM levels in *A. hydrophila*-infected HKM and the inhibitory role of BAPTA/AM, EGTA, nifedipine and verapamil suggested CaM elevation to be Ca^2+^-dependent. Our studies with CaM-specific siRNA and the CaM inhibitor calmidazolium chloride demonstrated CaM to be pro-apoptotic that initiated the downstream expression of CaMKII*g*. Using the CaMKII*g*-targeted siRNA, specific inhibitor KN-93 and its inactive structural analogue KN-92 we report CaM-CaMKII*g* signalling to be critical for apoptosis of *A. hydrophila*-infected HKM. Inhibitor studies further suggested the role of calpain-2 in CaMKII*g* expression. CaMK Kinase (CaMKK), the other CaM dependent kinase exhibited no role in *A. hydrophila*-induced HKM apoptosis. We report increased production of intracellular cAMP in infected HKM and our results with KN-93 or KN-92 implicate the role of CaMKII*g* in cAMP production. Using siRNA to PKACA, the catalytic subunit of PKA, anti-PKACA antibody and H-89, the specific inhibitor for PKA we prove the pro-apoptotic involvement of cAMP/PKA pathway in the pathogenicity of *A. hydrophila*. Our inhibitor studies coupled with siRNA approach further implicated the role of cAMP/PKA in activation of extracellular signal-regulated kinase 1 and 2 (ERK 1/2). We conclude that the alteration in intracellular Ca^2+^ levels initiated by *A. hydrophila* activates CaM and calpain-2; both pathways converge on CaMKII*g* which in turn induces cAMP/PKA mediated ERK 1/2 phosphorylation leading to caspase-3 mediated apoptosis of infected HKM.

## Author Summary

*Aeromonas hydrophila* is a natural fish pathogen and is known to induce apoptosis of HKM. Head kidney is an important immune-organ in fish and HKM are critical for immunity against the invading pathogen. The mechanisms of cell death induced by *A. hydrophila* are incompletely characterized. We have studied the role of Ca^2+^-dependent signalling pathways in the induction of *A. hydrophila*-induced HKM apoptosis. We observed that *A. hydrophila* infection led to increased CaM expression in infected HKM which was Ca^2+^-dependent. The inhibitor and siRNA studies suggested CaM to be pro-apoptotic and triggered CaMKII*g* expression in the infected HKM. Calpain-2 appeared to influence CaMKII*g* expression. However, further studies are needed to understand the process. We report that the CaM-CaMKII*g* pathway is important for initiating cAMP production within the infected HKM. The pro-apoptotic activation of cAMP dependent PKA was quite evident. The activation of ERK 1/2 was observed in the HKM and results clearly suggested the pro-active role of cAMP/PKA in the process. Thus we conclude that CaM-CaMKII*g* initiates the cAMP/PKA pathway that induces ERK 1/2 phosphorylation to promote caspase-3 mediated apoptosis of the *A. hydrophila*-infected HKM.

## Introduction

*Aeromonas hydrophila*, a Gram-negative, rod-shaped, facultatively intracellular bacterium is commonly found as part of the normal microbial flora in the aquatic environment [1]. The pathogenicity of *A. hydrophila* is complex and multi-factorial. It induces a plethora of symptoms in fish characterized by severe open dermal ulcers, anaemia, visceral granulomata, septicaemia, failure of osmoregulatory balance and death, which together comprise the ulcerative disease syndrome or UDS [2]. *A. hydrophila* is also known for its wide range of host tropism that includes amphibians, reptiles as well as mammals [2]. In humans, this bacterium is frequently associated with individuals suffering from gastroenteritis, wound infections, septicemia and immunodeficiency disorders [1].

Pathogen-induced alterations in intracellular Ca^2+^ with varied effects have been well documented. In several instances an alteration in the intracellular Ca^2+^-levels was found to be a pre-requisite for pathogen-induced apoptosis of different cell types including macrophages [3]. Studies have also documented that initiation of Ca^2+^-influx delays the onset of apoptosis facilitating pathogen survival and growth inside the host macrophages [4]. In this context, it is worth mentioning that all such observations were based on studies in the mammalian systems and that there is scarcely any information on the role of Ca^2+^ or its dependent kinases in host-pathogen interactions in fish.

Calmodulin is one of the most abundant and well characterised Ca^2+^ sensor proteins [5]. It regulates numerous Ca^2+^-mediated cellular functions such as cell growth, differentiation, proliferation and apoptosis in divergent models including macrophages [6]. The increased cytosolic Ca^2+^ binds to CaM and the resulting Ca^2+^-CaM interaction leads to activation of several protein kinases including CaM-dependent kinases (CaMKs) [7]. The CaMK cascade includes CaMKII and CaMKK. CaMKII is one of the best characterised of CaMK family of kinases. It has been suggested that CaMKII, a serine/threonine kinase, is activated by Ca^2+^-CaM binding, followed by rapid autophosphorylation on Thr^286^, which abolishes its auto-inhibition [8]. As a consequence, a transient elevation in Ca^2+^ leads to a prolonged activation of CaMKII. Among the CaMKII isoforms, the gamma-isoform of CaMKII (CaMKII*g*) has been reported in macrophage functioning [6], [9] and implicated both as pro- [6] and anti-apoptotic [10] in different studies.

The cyclic nucleotide, cAMP, is generated from ATP by adenylyl cyclases (ACs), whereas the phosphodiesterases (PDEs) catalyse its hydrolytic degradation. In eukaryotic cells cAMP requires the protein kinase A complex (PKA) as an intermediate to carry out its effects. Studies indicate that cAMP/PKA-pathway regulates a broad range of cellular responses that includes its central role as both a pro- and anti-apoptotic regulator [11]. There are varied pathways by which cAMP/PKA mediates its action and activation of MAPKs is one of them [12]. The MAPK family is composed of the extracellular signal-regulated kinase 1 and 2 (ERK 1/2), p38 and stress activated protein kinase/c-Jun N-terminal kinase (SAPK/JNK) pathways [13]. This family of kinases is important in a wide spectrum of cellular functions like proliferation, cytokine biosynthesis, cytoskeletal organization [14]. Amongst the MAPK family, activation of ERK 1/2 in pathogen-induced macrophage apoptosis is well characterized [15].

Mononuclear phagocytes such as macrophages are essential to the innate immune response against invading microorganisms. It has been suggested that one mechanism by which *A. hydrophila* induces pathogenicity in fish is through initiation of host macrophage apoptosis. In fish, especially teleosts, head kidney (HK) represents the anterior part of kidney. It is analogous to the mammalian bone marrow and the primary site of definitive hematopoiesis. The HK appears in the pre-hatching embryos on either side of the pharynx as renal units, each with a single glomerulus and short renal tubule. However, during the course of development the renal elements get degraded and it becomes primarily involved in hematopoiesis, antibody production and regulating immune-endocrine axis [16], [17]. The HKM serves as the first line of defence against invading pathogens. We have shown that HKM are crucial in the pathogenesis of *A. hydrophila* at the cellular level [18]. Recently, we demonstrated that live *A. hydrophila* infection leads to apoptotic death of HKM, involving calpain activation [19]. Here, we have identified and characterized other signalling effectors and executors that are involved in *A. hydrophila*-HKM interactions. *Clarias gariepinus* was selected as our model because of its availability round the year, ability to adapt to laboratory conditions and for having easily identifiable immune organs.

## Results

### CaM Expression Is Primal for *A. hydrophila*-Induced HKM Apoptosis

We earlier reported *A. hydrophila*-induced intracellular Ca^2+^ influx and subsequent calpain-2 activation in the infected HKM [19]. This study was designed to identify the role of other key molecules of Ca^2+^-pathway on *A. hydrophil*a-induced HKM apoptosis and CaM was a rational candidate. At the outset, we checked for CaM expression at mRNA level following infection with *A. hydrophila*. In the absence of genome sequence of *C. gariepinus* degenerate primers for CaM were designed using the homologous stretch across vertebrates as the template. The PCR product was cloned and sequenced and the sequence showed 100% identity with CaM-mRNA sequence of channel catfish, *Ictalurus* sp. Primers for real time analysis were designed from this sequence and the real-time data revealed maximum induction of CaM at 2 h post-infection (p.i.) at the mRNA level (P<0.05) (Figure S1A). The next step was to check *A. hydrophila*-induced CaM protein expression using CaM assay kit. We observed maximum CaM protein expression at 2 h p.i. (P<0.05) (Figure S1B) and hence selected this time point for subsequent studies on CaM. At further time points CaM mRNA and protein levels decreased significantly reaching basal levels at 24 h p.i. (data not shown).

**Figure 1:**
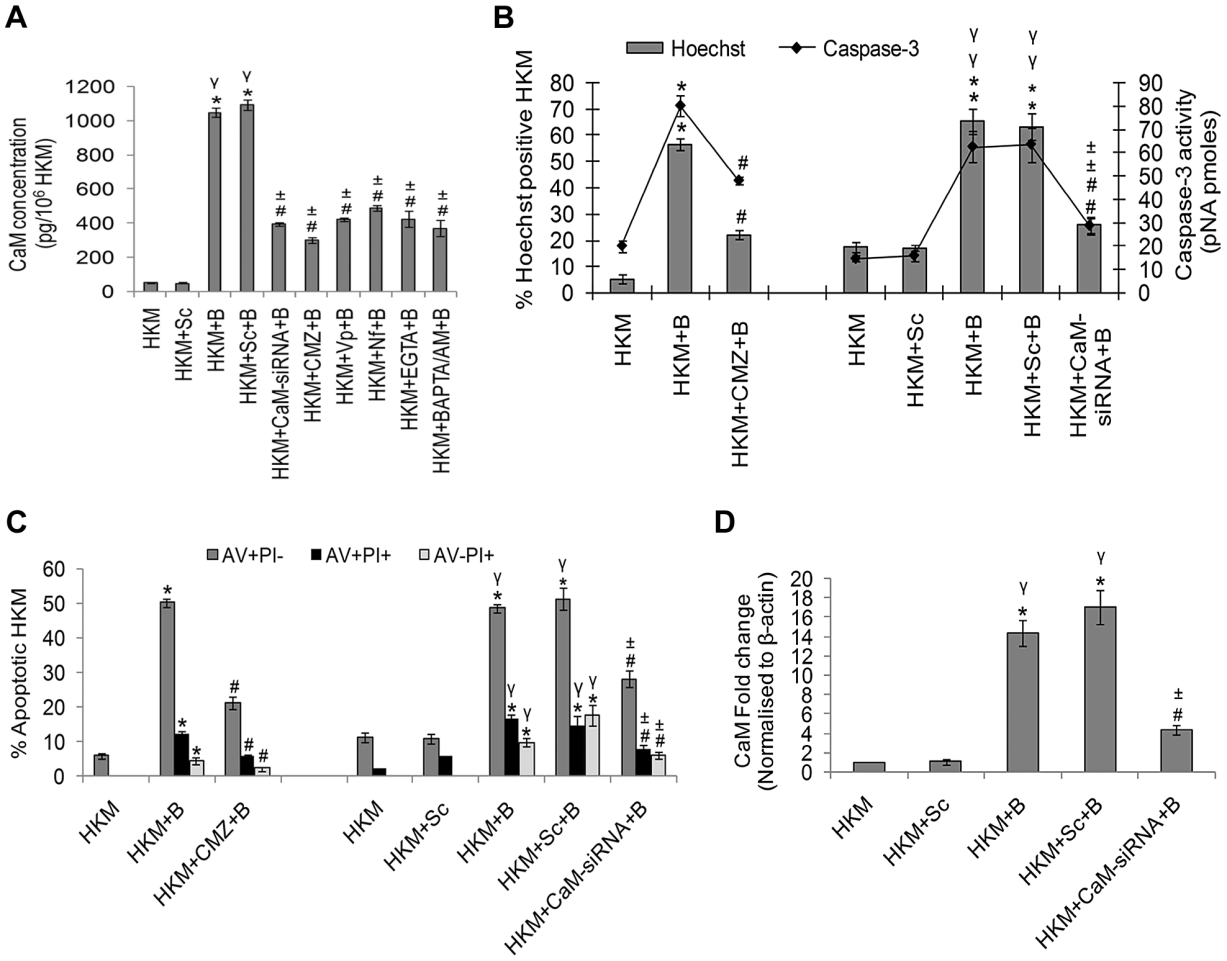
*A. hydrophila* infection leads to increased CaM expression in HKM. (A) HKM transfected separately with CaM-siRNA, scrambled siRNA or pre-treated with CMZ, Vp, Nf, EGTA, BAPTA/AM for different time periods were infected with *A. hydrophila* and CaM protein content measured in the lysates 2 h p.i. using EIA kit. HKM pre-treated with CMZ or transfected with CaM-siRNA or scrambled siRNA were infected with *A. hydrophila* and checked for (B) Hoechst 33342 positive cells and caspase-3 activity and (C) AV-PI staining 24 h p.i. (D) HKM transfected with CaM-siRNA or scrambled siRNA were infected with *A. hydrophila* and CaM mRNA expression detected by real time PCR 2 h p.i. Vertical bars represent mean ± SE (n = 6). *P<0.05, compared to HKM; **^γ^**P<0.05, compared to HKM+Sc; **^#^**P<0.05, compared to HKM+B; **^±^**P<0.05, compared to HKM+Sc+B. HKM, control head kidney macrophage; HKM+Sc, HKM transfected with scrambled siRNA; HKM+B, HKM infected with *A. hydrophila*; HKM+Sc+B, HKM transfected with scrambled siRNA infected with *A. hydrophila*; HKM+CaM-siRNA+B, HKM transfected with CaM-siRNA infected with *A. hydrophila*; HKM+BAPTA/AM+B, HKM pre-treated with BAPTA/AM infected with *A. hydrophila*; HKM+EGTA+B, HKM pre-treated with EGTA infected with *A. hydrophila*; HKM+Nf+B, HKM pre-treated with Nf infected with *A. hydrophila*; HKM+Vp+B, HKM pre-treated with Vp infected with *A. hydrophila*; HKM+CMZ+B, HKM pre-treated with CMZ infected with *A. hydrophila*.

CaM activity was assayed in presence of the CaM antagonist-CMZ. This CaM antagonist binds reversibly to CaM thus inhibiting CaM-mediated enzyme activation [20]. Pre-treatment with CMZ inhibited CaM activation (P<0.05) in infected HKM (Figure 1A) which clearly suggested the role of CaM on *A. hydrophila*-induced HKM-pathology. Further, pre-treatment with Ca^2+^-chelators BAPTA/AM, EGTA and the Ca^2+^-channel blockers Nf and Vp led to significant reduction in CaM activity at the protein levels indicating CaM expression to be Ca^2+^-dependent in the infected HKM (Figure 1A).

Our next step was to correlate CaM with HKM apoptosis. The HKMs were pre-treated with CMZ and apoptosis assessed 24 h p.i. by Hoechst, AV-PI staining and caspase-3 activation using specific assay kit. Pre-treatment with CMZ inhibited (P<0.05) apoptotic death and attenuated caspase-3 activity (P<0.05) (Figure 1B and 1C) in infected HKM implicating the role of CaM on initiating *A. hydrophila*-induced HKM apoptosis. These results were also confirmed using CaM-siRNA. Transfection with CaM-siRNA down-regulated CaM expression at mRNA level (Figure 1D), protein level (Figure 1A) at 2 h p.i. and also attenuated *A. hydrophila*-induced HKM apoptosis at 24 h p.i. (Figure 1B and 1C). Our results, for the first time, indicated the pro-apoptotic role of CaM in *A. hydrophila*-induced HKM apoptosis.

### CaMKII*g* Is the Likely CaM-Dependent Kinase Inducing HKM Apoptosis

**Figure 2:**
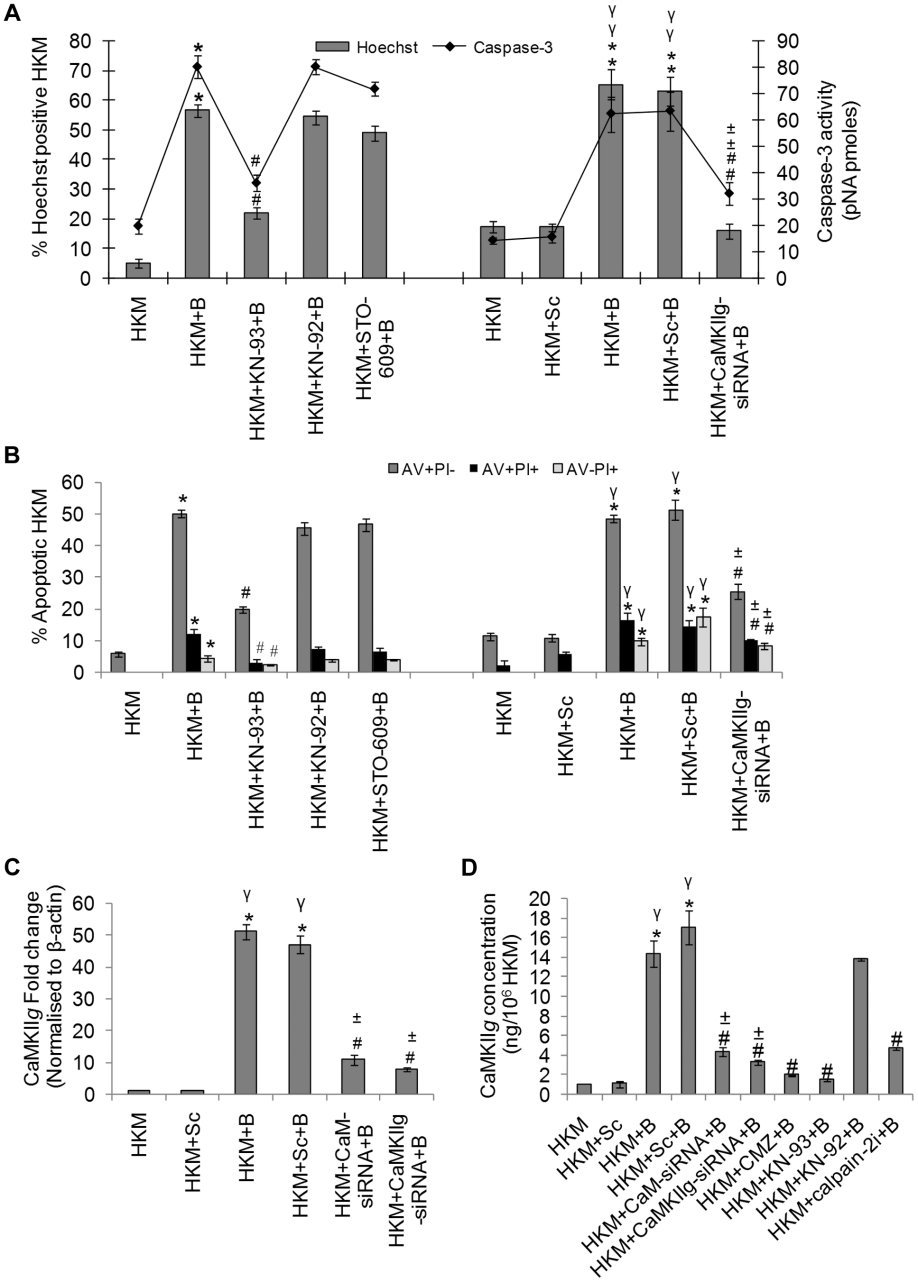
CaMKII*g* is CaM-dependent and pro-apoptotic. HKM were pre-treated separately with KN-93, KN-92, STO-609 or transfected with CaMKII*g*-siRNA or scrambled siRNA then infected with *A. hydrophila* and checked for (A) Hoechst 33342 positive cells and caspase-3 activity and (B) AV-PI staining 24 h p.i. (C) HKM were transfected separately with CaM-siRNA, CaMKII*g*-siRNA or scrambled siRNA then infected with *A. hydrophila* and CaMKII*g* mRNA expression detected 2 h p.i. by real time PCR. (D) HKM were transfected with CaM-siRNA, CaMKII*g*-siRNA, scrambled siRNA or pre-treated with CMZ, KN-93, KN-92, calpain-2*i* for different time periods then infected with *A. hydrophila* and CaMKII*g* protein content detected 24 h p.i. using EIA kit. Vertical bars represent mean ± SE (n = 6). *P<0.05, compared to HKM; **^γ^**P<0.05, compared to HKM+Sc; **^#^**P<0.05, compared to HKM+B; **^±^**P<0.05, compared to HKM+Sc+B. HKM, control head kidney macrophage; HKM+Sc, HKM transfected with scrambled siRNA; HKM+B, HKM infected with *A. hydrophila*; HKM+Sc+B, HKM transfected with scrambled siRNA infected with *A. hydrophila*; HKM+CaM-siRNA+B, HKM transfected with CaM-siRNA infected with *A. hydrophila*; HKM+CaMKII*g*-siRNA+B, HKM transfected with CaMKII*g*-siRNA infected with *A. hydrophila*; HKM+CMZ+B, HKM pre-treated with CMZ infected with *A. hydrophila*; HKM+KN-93+B, HKM pre-treated with KN-93 infected with *A. hydrophila*; HKM+KN-92+B, HKM pre-treated with KN-92 infected with *A. hydrophila*; HKM+STO-609+B, HKM pre-treated with STO-609 infected with *A. hydrophila*; HKM+calpain-2*i*+B, HKM pre-treated with calpain-2*i* infected with *A. hydrophila.*

Among several downstream events that are initiated due to altered CaM activity the activation of CaMKs are believed to be important. We studied the role of CaMKK and CaMKII, two important CaMKs in our model. The HKM were pre-treated with specific pharmacological inhibitors of CaMKK and CaMKII and apoptosis was evaluated 24 h p.i. by Hoechst and AV-PI staining. First, we used the CaMKK inhibitor STO-609 which specifically inhibits the CaMKK activity by suppressing Ca^2+-^dependent signalling [21]. STO-609 had no preventive effect on *A. hydrophila*-induced HKM apoptosis (Figure 2A and 2B). Subsequently, we used the CaMKII specific inhibitor KN-93 and its structural analogue KN-92 to investigate the role of CaMKII on the process. KN-93 competitively blocks CaM binding to the kinase whereas KN-92 is a congener of KN-93 without CaM kinase inhibitory activity and is used as an experimental control [22]. We observed that pre-treatment with KN-93 attenuated caspase-3 activity and conferred significant protection to the infected HKM from *A. hydrophila*-induced apoptosis (Figure 2A and 2B). The inactive analogue KN-92 failed to inhibit HKM apoptosis suggesting a mediatory role of CaMKII on initiating apoptosis of infected HKM.

To validate these observations qRT-PCR and EIA were done to evaluate the status of the specific transcript and protein. Degenerate primers for CaMKII*g* were designed using the homologous stretch across vertebrates as the template. The PCR product was cloned, sequenced and the sequence showed 100% identity with CaMKII*g* of zebrafish, *Danio rerio*. Primers for real time analysis were designed from this sequence for quantifying CaMKII*g* levels. We observed that *A. hydrophila* infection led to significant increase in CaMKII*g* mRNA expression with maximum levels being at 2 h p.i. (Figure S2A), which decreased thereafter reaching basal levels at 24 h p.i. (data not shown). We checked CaMKII*g* concentrations using specific EIA kit 24 h p.i. as CaMKII is detected for prolonged period even after the Ca^2+^ signal has decayed [8]. A significant increase in CaMKII*g* levels (P<0.05) was noted in the infected HKM which was inhibited (P<0.05) in presence of CMZ and KN-93, antagonists to CaM and CaMKII*g* respectively (Figure 2D) but not by KN-92 in *A. hydrophila*-infected HKM. Using siRNAs specific to CaM and CaMKII*g* that significantly down-regulated CaMKII*g* at mRNA and protein levels (Figure 2C and 2D), significant attenuation of HKM apoptosis was also observed (Figure 2A and 2B). Taken together our data implicates Ca^2+^-induced CaM-CaMKII*g* expression to be a critical event in *A. hydrophila*-induced HKM apoptosis.

### CaM-CaMKII-Induced Activation of cAMP/PKA Pathway Is a Prime Event in *A. hydrophila*-Induced HKM Apoptosis

**Figure 3:**
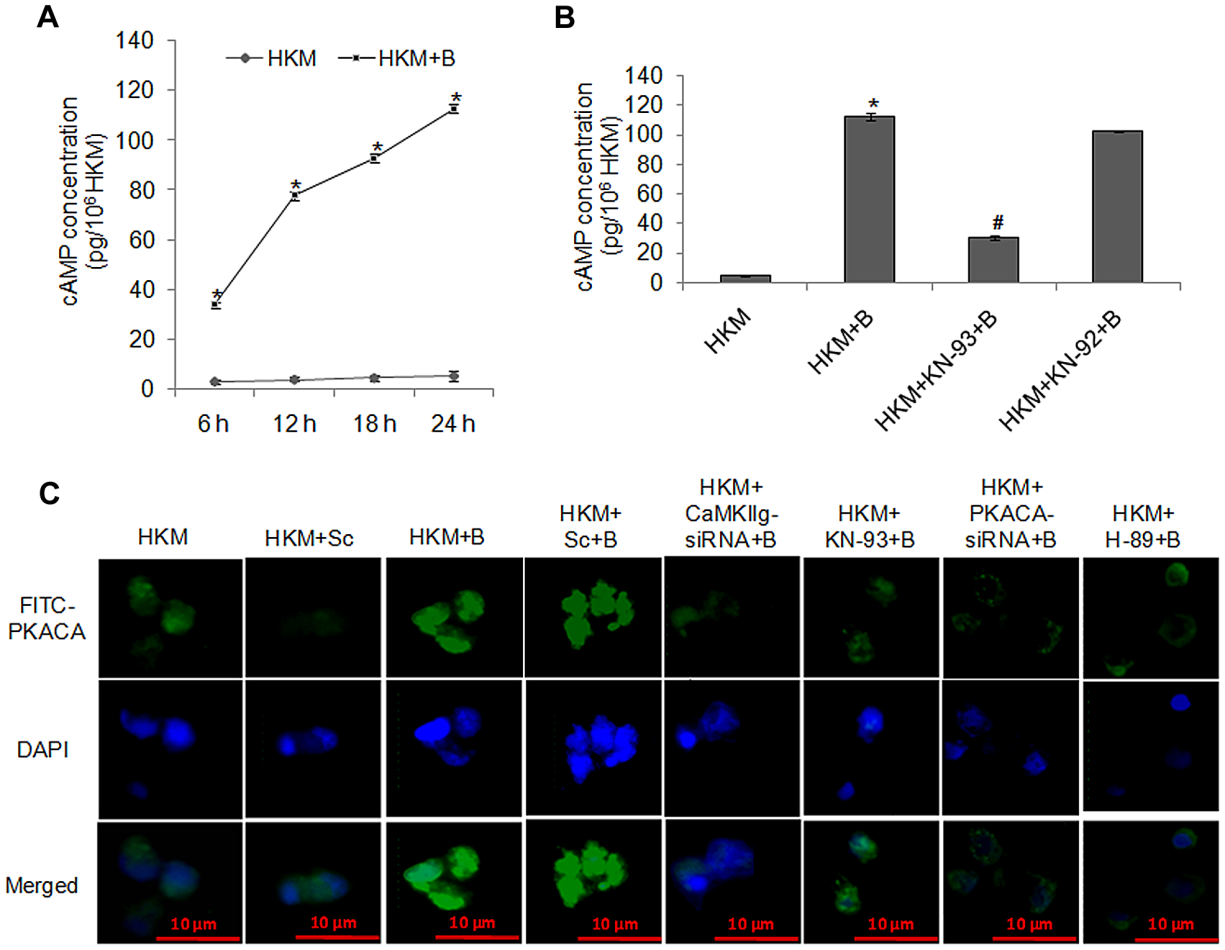
*A. hydrophila*-infection triggers cAMP release, induces activation and nuclear translocation of PKACA. (A) Cell lysates of HKM infected with *A. hydrophila* were checked for intracellular cAMP release at indicated time point p.i. using EIA kit. (B) HKM pre-treated with KN-93 or KN-92 were infected with *A. hydrophila* and cAMP release was measured in the cell lysates at 24 h p.i. (C) HKM were transfected with CaMKII*g*-siRNA, PKACA-siRNA or scrambled siRNA or pre-treated with KN-93 or H-89 then infected with *A. hydrophila* and PKACA activation and nuclear translocation checked by immunofluorescence. The images are representative of three independent experiments. Vertical bars represent mean ± SE (n = 6). *P<0.05, compared to HKM; **^#^**P<0.05, compared to HKM+B. HKM, control head kidney macrophage; HKM+Sc, HKM transfected with scrambled siRNA; HKM+B, HKM infected with *A. hydrophila*; HKM+Sc+B, HKM transfected with scrambled siRNA infected with *A. hydrophila*; HKM+CaMKII*g*-siRNA+B, HKM transfected with CaMKII*g*-siRNA infected with *A. hydrophila*; HKM+PKACA-siRNA+B, HKM transfected with PKACA-siRNA infected with *A. hydrophila*; HKM+KN-93+B, HKM pre-treated with KN-93 infected with *A. hydrophila*; HKM+KN-92+B, HKM pre-treated with KN-92 infected with *A. hydrophila*; HKM+H-89+B, HKM pre-treated with H-89 infected with *A. hydrophila*.

We studied cAMP/PKA signalling in *A. hydrophila*-induced HKM apoptosis as several macrophage activities appear to be controlled by this pathway [23]. Lysates from infected HKM were collected at different time intervals and assayed for intracellular cAMP production. It was observed that *A. hydrophila*-infection led to significant increase in intracellular cAMP levels at all time points studied with maximum production recorded at 24 h p.i. (Figure 3A). The cell-permeable cAMP analogue (8-Br-cAMP) when used as positive control elevated intracellular cAMP level and induced HKM apoptosis (data not shown). Our results thus implicate the role of cAMP in the pathophysiology associated with *A. hydrophila* infection.

We next studied the role of CaMKII*g* on initiating the production of cAMP in infected HKM since Ca^2+^-dependent signalling molecules often converge on cAMP [24]. As the cAMP levels were maximally induced at 24 h p.i., this time point was chosen for subsequent studies. It is evident from Figure 3B that *A. hydrophila*-induced cAMP levels were significantly reduced following pre-treatment with CaMKII specific inhibitor-KN-93. This suggests CaM-activated CaMKII*g* plays an important role in cAMP generation in the infected HKM.

Protein kinase A is an important mediator of the cAMP dependent signalling pathway. The cyclic nucleotide cAMP causes PKA activation by binding to the regulatory subunit, triggering release, activation and nuclear translocation of PKACA [25], [26]. Therefore, our next step was establishing the involvement of PKACA in the pathogenicity of *A. hydrophila*. Degenerate primers specific for PKACA were designed the PCR product cloned, sequenced and nBLAST analysis suggested 100% identity with PKACA of *Ictalurus* sp. We used real time primers based on the sequence obtained and observed that *A. hydrophila*-infection induced a several fold increase in PKACA transcripts with maximum expression recorded by 2–6 h p.i. (P<0.05) (Figure S2B) and thereafter it started declining (data not shown) though significant level of PKACA mRNA expression was noted till 24 h p.i. This decline in PKACA transcripts at later time points probably serves as controlling mechanism to prevent the overshoot of cAMP dependent downstream events in the infected cells.

**Figure 4:**
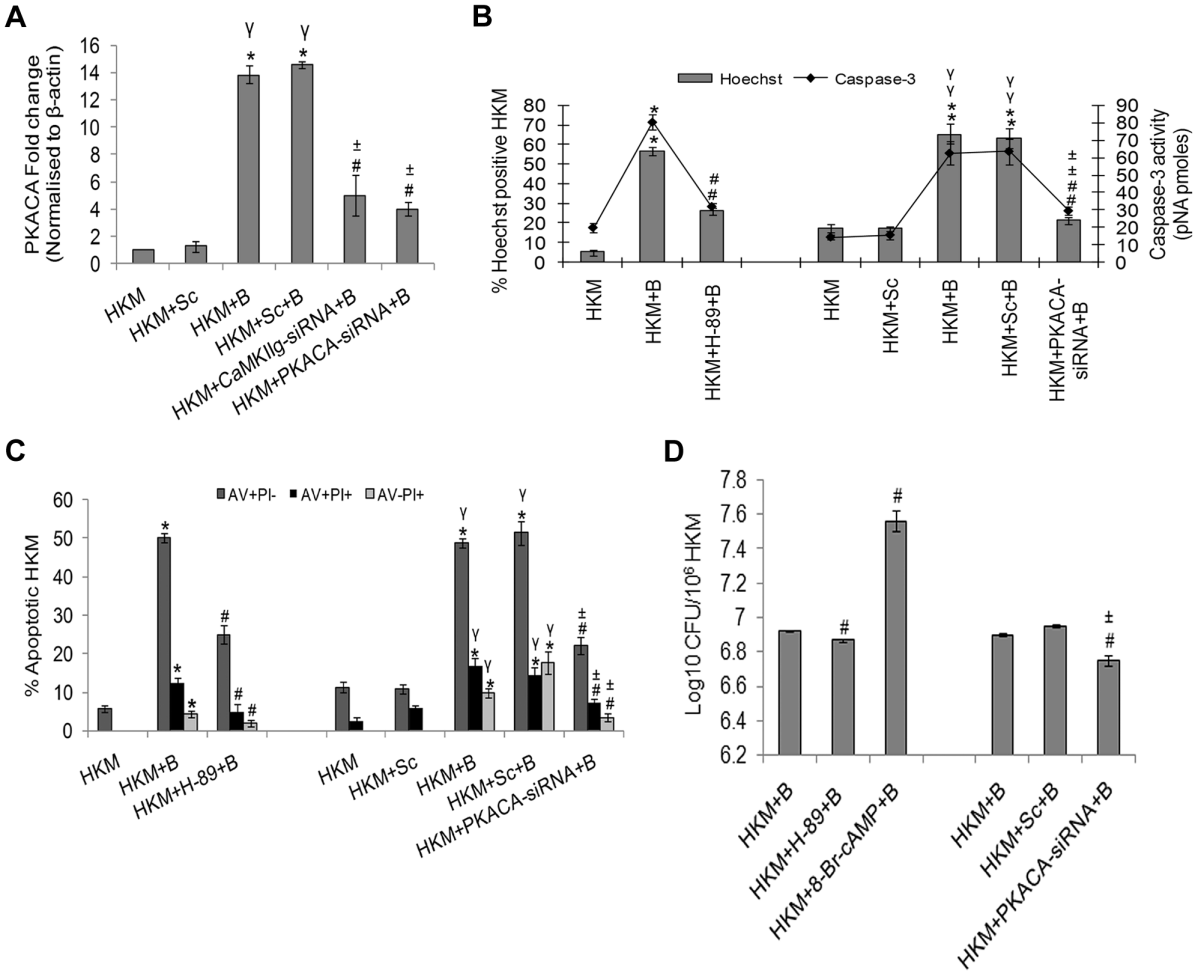
PKA is pro-apoptotic in *A. hydrophila*-infected HKM. (A) HKM were transfected with CaMKII*g*-siRNA, PKACA-siRNA or scrambled siRNA then infected with *A. hydrophila* and PKACA mRNA expression detected by real time PCR 2 h p.i. HKM were pre-treated with H-89 or transfected with PKACA-siRNA or scrambled siRNA then infected with *A. hydrophila* and checked for (B) Hoechst 33342 positive cells and caspase-3 activity and (C) AV-PI staining 24 h p.i. (D) *A. hydrophila* viability was checked following either pre-incubation of HKM with H-89, co-incubation with 8-Br-cAMP or transfection with PKACA-siRNA. The intracellular bacterial number was determined by dilution plating on nutrient agar plate. Vertical bars represent mean ± SE (n = 6). *P<0.05, compared to HKM; **^γ^**P<0.05, compared to HKM+Sc; **^#^**P<0.05, compared to HKM+B; **^±^**P<0.05, compared to HKM+Sc+B. HKM, control head kidney macrophage; HKM+Sc, HKM transfected with scrambled siRNA; HKM+B, HKM infected with *A. hydrophila*; HKM+Sc+B, HKM transfected with scrambled siRNA infected with *A. hydrophila*; HKM+CaMKII*g*-siRNA+B, HKM transfected with CaMKII*g*-siRNA infected with *A. hydrophila*; HKM+PKACA-siRNA+B, HKM transfected with PKACA-siRNA infected with *A. hydrophila*; HKM+H-89+B, HKM pre-treated with H-89 infected with *A. hydrophila*; HKM+8-Br-cAMP+B, HKM exposed to 8-Br-cAMP infected with *A. hydrophila*.

CaMKII*g* and PKACA targeted siRNAs also led to significant inhibition of PKACA at the transcript level (Figure 4A). We then studied PKACA expression and nuclear translocation by immunofluorescence 24 h p.i. in infected HKM. The results with anti-PKACA antibody clearly suggested increased PKACA expression in nuclei of *A. hydrophila*-infected HKM (Figure 3C).

We extended the study by including PKA specific inhibitor H-89, CaMKII specific inhibitor-KN-93 or transfected the HKM with CaMKII*g*-siRNA and PKACA-siRNA. The inhibitor H-89 blocks PKA actions through competitive inhibition of the adenosine triphosphate (ATP) site on the PKA catalytic subunit [27]. We observed decreased expression and restricted nuclear movement of PKACA in the infected HKM at 24 h p.i. in presence of H-89, KN-93, CaMKII*g*-siRNA and PKACA-siRNA (Figure 3C). Our results for the first time established the role of CaMKII*g* on PKACA activation in *A. hydrophila*-infected HKM. The presence of H-89 and PKACA-siRNA also led to significant alleviation (P<0.05) of apoptotic death and diminished caspase-3 activity in the infected HKM (Figure 4B and 4C) suggesting the critical role of PKA pathway on the pathogenicity induced by the bacteria.

We next studied the link between the activation of PKA and intra-cellular survival of *A. hydrophila*. Pre-incubation of HKM with H-89 or PKACA-siRNA reduced (P<0.05) recovery of intracellular bacteria (Figure 4D). Besides, we found that H-89 addition till 60 mins p.i. reduced the bacterial load and beyond that exhibited little inhibitory effect (Figure S3). No direct effect of H-89 on *A. hydrophila* was observed as adding the inhibitor at the particular concentration did not affect the viability, growth and initial uptake by HKM (data not shown).

### ERK 1/2 Activation Is Pro-Apoptotic in *A. hydrophila* Infection and Depends on cAMP/PKA Pathway

**Figure 5:**
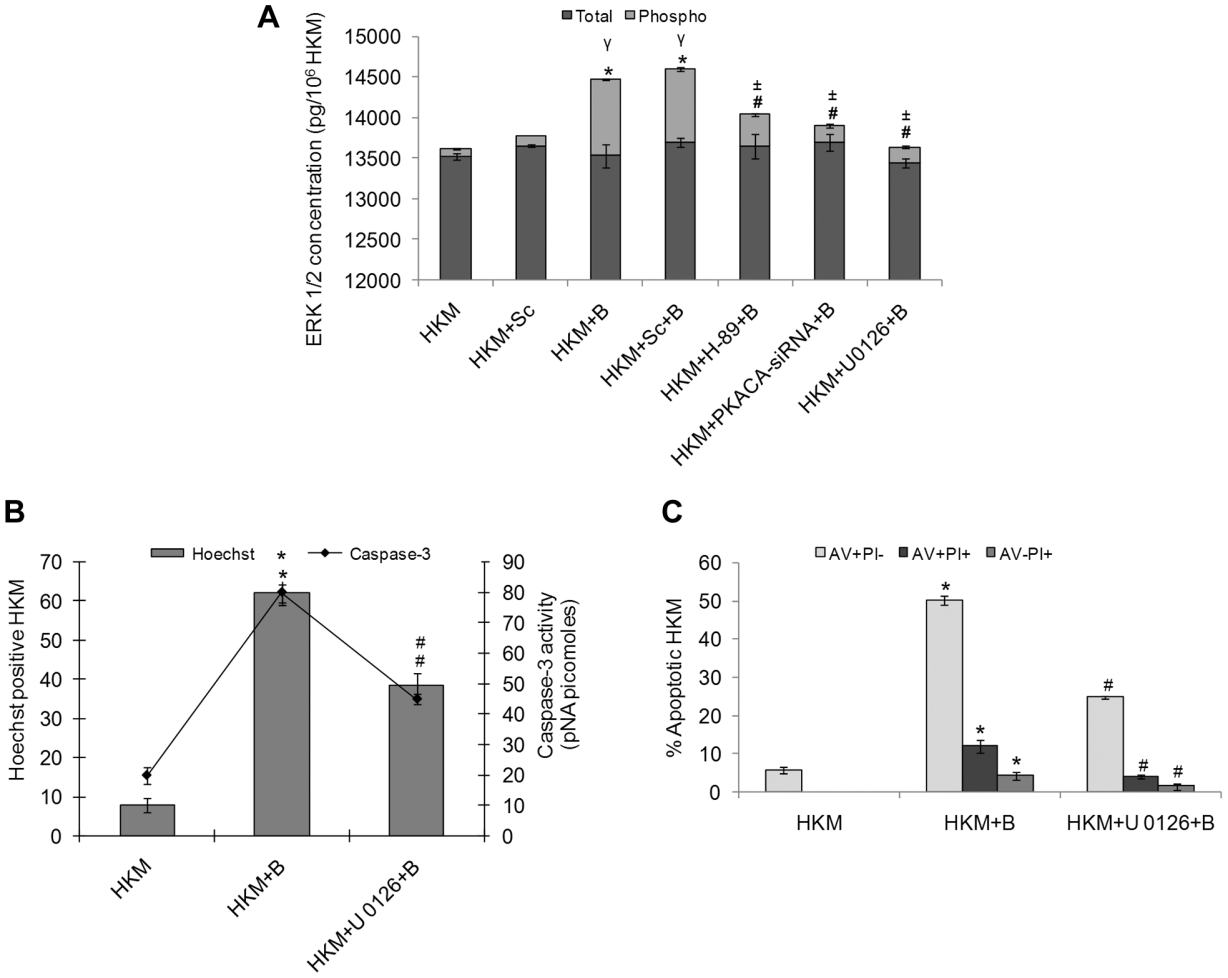
*A. hydrophila*-induced ERK 1/2 phosphorylation is PKA mediated and is pro-apoptotic in HKM. (A) HKM were transfected with PKACA-siRNA, scrambled siRNA or pre-treated with H-89, U 0126 then infected with *A. hydrophila* and checked for total ERK and pERK activity 24 h p.i. using EIA based kits. HKM were pre-treated with or without U 0126 then infected with *A. hydrophila* and checked for (B) Hoechst 33342 positive cells and caspase-3 activity and (C) AV-PI staining 24 h p.i. Vertical bars represent mean ± SE (n = 6). *P<0.05, compared to HKM; **^γ^**P<0.05, compared to HKM+Sc; **^#^**P<0.05, compared to HKM+B; **^±^**P<0.05, compared to HKM+Sc+B. HKM, control head kidney macrophage; HKM+Sc, HKM transfected with scrambled siRNA; HKM+B, HKM infected with *A. hydrophila*; HKM+Sc+B, HKM transfected with scrambled siRNA infected with *A. hydrophila*; HKM+PKACA-siRNA+B, HKM transfected with PKACA-siRNA infected with *A. hydrophila*; HKM+H-89+B, HKM pre-treated with H-89 infected with *A. hydrophila*; HKM+U 0126+B, HKM pre-treated with U 0126 infected with *A. hydrophila*.

The ERK 1/2 pathway has been implicated both as pro-and anti-apoptotic in host macrophage responses. We aimed to investigate the involvement of ERK 1/2 activation in *A. hydrophila*-induced HKM apoptosis. The HKM were infected with *A. hydrophila* in presence or absence of specific inhibitors, lysed at indicated time intervals and the changes in the levels of total and phosphorylated ERK 1/2 measured using specific EIA kits. It is evident from Figure 5A that there was significant increase in pERK 1/2 levels in the *A. hydrophila*-infected HKM though no noticeable change was observed in total-ERK 1/2 levels. The role of cAMP/PKA on ERK 1/2 activation has been reported [28]. To investigate this possibility the HKM were pre-treated with PKA specific inhibitor H-89 and changes in total and pERK 1/2 levels studied. We observed that H-89 significantly inhibited pERK 1/2 levels in the infected HKM implicating the role of cAMP/PKA pathway in initiating the kinase activity. U0126 is a non-ATP competitive inhibitor of mitogen-activated protein kinase kinase (MEK) which exerts its effect by inhibiting the ability of MEK to phosphorylate downstream ERK 1/2 [29]. Hence it served as an effective negative control in the study. To further substantiate the role of cAMP/PKA pathway on ERK 1/2 activation we used PKACA-siRNA and our results (Figure 5A) confirmed ERK 1/2 activation to be downstream to cAMP/PKA in *A. hydrophila*-infected HKM.

We next investigated the contribution of ERK 1/2 activation on HKM apoptosis. For that HKM were pre-treated with or without U 0126 prior to infection with *A. hydrophila* and checked for apoptotic death using Hoechst 33342, AV-PI staining and caspase-3 activity 24 h p.i. It is clear from Figure 5B and 5C that inhibition of ERK 1/2 significantly inhibited (P<0.05) *A. hydrophila*-induced HKM apoptosis and lowered caspase-3 activity. Taken together our observations suggest ERK 1/2 activation is downstream event of cAMP/PKA pathway and is pro-apoptotic in *A. hydrophila*-infected HKM.

## Discussion

Calmodulin plays a significant role in microbial pathogenicity. Its role has been implicated in the pathogenicity of *Mycobacterium sp*. [28], *Clostridium perfingens* [30] and for survival of *Pneumocystis*-infected alveolar macrophages [31]. The presence of well conserved CaM is well documented in fish [32], [33]. As we observed increased intracellular Ca^2+^-levels in infected HKM [19] and CaM being a well-known Ca^2+^-sensor, we hypothesised a role of CaM on the pathogenicity of *A. hydrophila*. Indeed, results obtained from qRT-PCR, specific EIA assays, siRNA and pharmacological inhibitors conclusively demonstrated the importance of Ca^2+^-induced CaM expression in the initiation of *A. hydrophila*-induced HKM apoptosis. The kinetics of CaM expression, both at transcript and protein level demonstrated maximum expression at 2 h p.i. This suggested CaM expression at protein level closely follows pattern at transcript level, a phenomenon observed for many signalling molecules [34]. To our knowledge, this is the first report that clearly documents the pro-apoptotic involvement of CaM in *A. hydrophila* infections. We had earlier reported the role of calpain-2 in *A. hydrophila*-induced HKM apoptosis. We questioned whether the two Ca^2+^-dependent molecules crosstalk to initiate HKM apoptosis. Contrary to our expectations we did not observe any crosstalk between calpain-2 and CaM activity (data not shown), which suggests two possibilities: (a) CaM is resistant to the lytic action of calpains [35] and (b) CaM and calpain activity are independent to each other in our model.

Although there is little information regarding the mechanisms of CaM involvement in apoptosis, there are reports suggesting pro-apoptotic roles of CaM-dependent enzymes including CaMKs [8]. We reasoned that downstream activation of CaMKs is important for the initiation of apoptosis and inhibiting the kinase activities would confer protection to the infected HKM. To investigate this, we investigated the roles of CaMKK and CaMKII in initiating *A. hydrophila*-induced HKM apoptosis. We selected these two kinases as they are conserved, well characterised, have wide tissue distribution including macrophages and work through distinct pathways regulating diverse biological functions [8]. Our inhibitor studies indicated CaMKII involvement and clearly ruled out the involvement of CaMKK in the process. This observation also ruled out the involvement of CaMKI and CaMKIV in the process as both kinases require upstream phosphorylation of CaMKK for getting activated [7]. CaMKII has been reported to be both pro-apoptotic [6] and anti-apoptotic [10]. To investigate the role of CaMKII in *A. hydrophila*-induced HKM apoptosis we selected the gamma-isoform, CaMKII*g* as it is largely reported in macrophage functioning [6], [9]. Use of siRNAs confirmed the involvement of CaMKII*g* and its modulation by CaM in HKM due to *A. hydrophila* infection. It is worth mentioning that there is little information on the involvement of CaMKII*g* in pathogen-induced apoptosis except for its role in the pathogenicity induced by *Mycobacterium sp*. [28]. These results clearly indicated CaMKII*g* to be important in Ca^2+^-mediated apoptotic signalling downstream of CaM in *A. hydrophila-*infected HKM.

CaMKs are considered to be the likely substrates for calpain proteolysis [35]. Calpain-mediated CaMKIV activation is well documented in the apoptosis of SH-SY5Y cells [36] and cerebellar granule cells [37]. In contrast, there is no report on the role of calpains on CaMKII*g* expression especially in fish macrophages. In this connection, it is worth mentioning that pre-treatment with calpain-2 inhibitor led to significant decline in CaMKII*g* expression in infected HKM. Our finding for the first time suggested the existence of an alternate or calpain-mediated pathway for CaMKII*g* expression in fish macrophages following *A. hydrophila*-infection. Further studies are needed to understand the crosstalk between CaMKII*g* and activated calpains.

The role of cAMP varies between the prokaryotic and eukaryotic system. In eukaryotes cAMP signalling is important in diverse biological processes such as metabolism, memory formation and apoptosis [38]. To begin with, we initially looked for the changes of cAMP and observed significant increase in cAMP levels following *A. hydrophila* infection. Earlier studies with *Brucella suis* [39], *Bordetella pertussis* [40], *Bacillus anthracis* [41], *Mycobacterium sp* [28], *Pseudomonas aeruginosa* [42] and *Porphyromonas gingivalis* [43] also reported increased cAMP production *via* different mechanisms implicating cAMP in the pathogenicity of these microbes. It is interesting to note that the levels of *A. hydrophila*-induced cAMP observed by us is comparable to those recorded in many of these microbes which make us believe that such an *A. hydrophila*-induced effect could also be important for the pathogenicity of this bacteria. Besides, we also observed a time-dependent increase in cAMP production following *A. hydrophila* infection which corroborates with the earlier reports of Chopra *et al*., (2000) [44] in which a gradual increase in cAMP production was seen in the murine macrophage cell line RAW 264.7, treated with the *A. hydrophila*-toxin Act. There are also reports suggesting cAMP to be both pro- and anti-apoptotic [11]. A time-dependent increase of cAMP levels in infected HKM suggested that downstream cAMP-dependent pathways ultimately determine the outcome of infection in infected cell. This also led us to the identification that elevated cAMP levels are indeed due to CaM-dependent CaMKII*g* activity in the *A. hydrophila*-infected HKM. Determining how the CaM-CaMKII*g* axis is connected with cAMP production would be an interesting direction of the future. Previous studies have suggested that some isoforms of AC can be activated by Ca^2+^-CaM-CaMK by phosphorylation of the serine residues on AC [45]. It remains to be seen if this is also true in our system.

On establishing the role of cAMP we looked for its downstream targets in the infected HKM. Protein kinase A being an important intermediary in cAMP-dependent signalling, was an attractive candidate. Our observations using qRT-PCR, siRNA, cAMP/PKA-specific inhibitor, H-89 and PKACA antibody suggested that activation of cAMP/PKA is a downstream consequence of CaMKII*g* activation. Pre-treatment with H-89 did not influence CaMKII*g* activation in the infected HKM (data not shown). The cAMP/PKA pathway attributed to the virulence of *A. hydrophila* as inhibition of PKA activity led to increased HKM viability with concomitant decline in the number of intracellular bacteria. If PKA activation is essential for survival of *A. hydrophila* inside the HKM at what stage of infection is it important? We suggest PKA activation is critical at initial stages of infection as inhibiting PKA activity at late stages of infection had little effect on the replication of intra-cellular *A. hydrophila*. Our observations are similar to those reported by Gross *et al*., (2003) [39] for *Brucella suis* and we believe that *A. hydrophila* alike *B. suis* initiates an early cAMP/PKA-mediated virulence trait that helps in eluding macrophage-microbicidal responses critical at the onset of infection. Although, it is not possible from this study to conclude on how *A. hydrophila* affects cAMP/PKA pathway; we speculate that the bacteria could be releasing toxins which directly phosphorylate ACs or inhibit the activation of PDEs leading to increased accumulation of the cyclic nucleotide inside the HKM.

The participation of MAPKs has been widely observed during pathogenic invasion [46]. The genes encoding the MAPK family proteins are well conserved and have been reported in fish to be regulating various downstream targets such as transcription factors and heat shock proteins [47]. The involvement of MAPK cascade is beginning to be understood in the pathogenicity of *A. hydrophila* in fish [48]. Amongst the MAPK family members, ERK 1/2 has been reported to act both as pro- and anti-apoptotic factor under different conditions of pathogenic-stress [46]. We sought to understand its role in *A. hydrophila*-infection and our results conclusively demonstrate pro-apoptotic role of ERK 1/2 in *A. hydrophila*-infected HKM. ERK 1/2 activation is also reported in *A. hydrophila* pathogenicity though in mammalian system [1]. Interestingly, we also noted pro-apoptotic role of JNK and p38, the other members of the MAPK family in initiating *A. hydrophila*-induced HKM apoptosis (unpublished observations). This suggests that *A. hydrophila* uses a common signalling mechanism to activate the MAPK cascade to induce apoptosis in different hosts. Thus, our observations contradict those reported in *Mycobacterium* [27], *Pseudomonas* [49] or *Yersinia* [50] where the differential activation of MAPK family was reported to be important for pathogenicity.

Caspases are members of a family of cysteine proteases that are involved in apoptosis induced by various stimuli in different cell types, including macrophages. We recently reported *A. hydrophila*-induced HKM apoptosis to be caspase-3 mediated [19]. Presently, we aimed to determine what other signals initiated out of Ca^2+^ wave have a role in the activation of caspase-3 following *A. hydrophila* infections. From the available literature it is evident that a relationship between MAPKs and caspase-3 exist with reports suggesting MAPK activating caspase-3 [51] and *vice versa* [52]. Interestingly, our results clearly showed caspase-3 to be downstream of MAPK (ERK 1/2) in *A. hydrophila*-infected HKM. The means by which MAPKs regulate caspase-3 activity remain unclear and further investigation in this direction will aid in understanding the pathogenicity of this bacteria.

**Figure 6:**
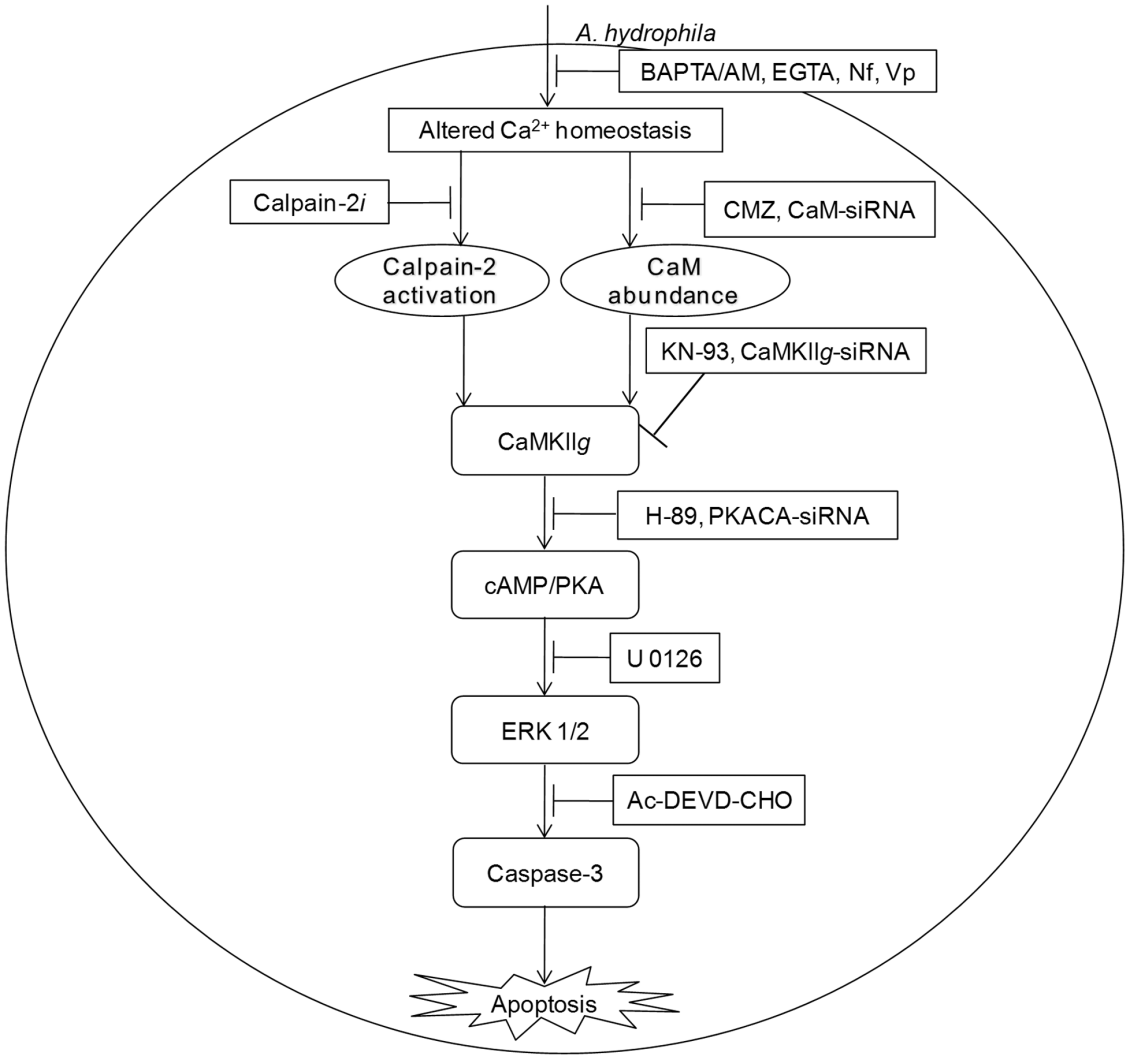
CaM-CaMKII-cAMP/PKA-ERK 1/2 axis leads to caspase-3 mediated apoptosis of *A. hydrophila*-infected HKM. *A. hydrophila*-elevated Ca^2+^ leads to downstream calpain-2 activation and CaM expression. The pathways converge at CaMKII*g* to induce cAMP/PKA mediated activation of ERK 1/2 leading to caspase-3 mediated apoptosis of *A. hydrophila*-infected HKM.

To conclude, our findings emphasise the unrecognized role of CaM in initiating *A. hydrophila*-induced HKM apoptosis. We propose that the Ca^2+^-influx initiated by *A. hydrophila* activates pro-apoptotic CaM and downstream CaMKII*g* in infected HKM. Calpains also appear to have some role on CaMKII*g* expression. CaMKII*g* in turn initiates a network of signals resulting in the downstream activation of cAMP/PKA and MAPK pathways respectively and the cascade of events culminate in caspase-3 activation and apoptosis of infected HKM (Figure 6).

## Materials and Methods

All the chemicals used in this study were purchased from Sigma unless otherwise stated.

### Ethics Statement

All animal experiments described in the present study were approved by the Animal Ethics Committee of University of Delhi (DU/ZOOL/IAEC-R/2013/33) and carried out in accordance with animal experimentation protocols approved by The Prevention of Cruelty to Animals Act, Govt. of India.

### Animal Care and Maintenance

Catfish (*Clarias gariepinus*, Siluriformes, 100±20 g) were maintained in 50-L glass tanks (2–3 fish per tank) under natural conditions. The water quality, dissolved oxygen content and pH were monitored regularly in each tank. The fish were fed boiled chicken liver *ad libitum* and acclimatized to laboratory conditions for 15 days before use for experimental purpose. During this period and when the experiments were conducted, fish health was routinely monitored by appearance and pathological examinations as mentioned earlier [53].

### Bacterial Preparations

The *A. hydrophila* (Strain 500297) was a gift from Dr. T. Ramamurthy, National Institute of Cholera and Enteric Diseases (NICED), India. The bacteria were grown to late log phase (12 h) in brain heart infusion broth (BHI, HiMedia) containing 100 μg mL^-1^ ampicillin at pH 7.4 overnight at 30°C with aeration and maintained in nutrient agar slants at 4°C. The pathogenicity of the strain was confirmed *in vitro* by hemolysin and cytotoxicity assays and by the ability to induce characteristic UDS lesions when injected into the fish [18].

### Isolation of HKM

Head kidneys were aseptically removed and placed in RPMI-1640 (Gibco-Invitrogen) with phenol-red indicator and supplemented with 25 mM HEPES (Gibco-Invitrogen) containing 1% penicillin-streptomycin (complete-RPMI). Single cell suspensions of each pair of head kidney were prepared using 100 μm wire mesh, diluted and the total cell population pelleted by centrifugation at 400×*g* for 10 mins at 4°C. The supernatant was discarded and the re-suspended pellet layered on discontinuous percoll density gradient (34/51) and centrifuged at 400×*g* for 20 mins at 4°C. The phagocyte rich fraction appearing above the 34/51 interface was collected, washed and enriched for macrophages as described earlier [19]. The purity of the HKM was checked by staining with Wright Giemsa Stain and viability determined using 0.4% trypan blue dye exclusion method.

### Infection Procedure

The HKM were seeded in 24 well plates. The *A. hydrophila* were grown to mid log phase, harvested and infected into HKM at a multiplicity of infection (MOI) of 50 for 60 mins. To kill extracellular bacteria, the infected HKM cultures were incubated for a further 1 h in RPMI containing 30 μg mL^−1^ chloramphenicol. Exposure to 30 μg mL^−1^ chloramphenicol for 1 h was sufficient to kill 100% of microorganisms but had no effect on macrophage viability. Uninfected HKM served as control and maintained in complete-RPMI for the same time.

The siRNA transfection was done using HiPerFect Transfection Reagent (Qiagen) as per manufacturer's instructions. The siRNA-Hiperfect complex (5 μl each) was mixed gently in Opti-MEM (Invitrogen) and incubated for 20 mins to allow complex formation. This complex was added to the isolated HKM maintained in Opti-MEM, mixed properly and incubated at 30°C and 5% CO_2_. Cell viability was continuously monitored and after 5 h of incubation, media was changed to complete-RPMI. At 24 h post-transfection, HKM were washed, placed in fresh complete-RPMI and infected with *A. hydrophila* as mentioned earlier and proceeded for apoptotic studies, qRT-PCR and protein assays to confirm knockdown. Targeted [(CaM, SENSE-5′-CCAUUACGACCAAAGAGUU-3′ and ANTISENSE-5′-AACUCUUUGGUCGUAAUGG-3′); (CaMKII*g*, SENSE-5′-GGACAUUUGGGCUUGUGGA-3′ and ANTISENSE-5′-UCCACAAGCCCAAAUGUCC-3′); (PKACA, SENSE-5′-CAGUAAAGGCUACAAUAAA-3′ and ANTISENSE-5′-UUUAUUGUAGCCUUUACUG-3′)] plus siCONTROL non-targeting siRNA pool (HS Number 29349990700) 5 nM each were used.

For inhibitor studies, HKM were pre-treated with or without 10 μM caspase-3 inhibitor (Acetyl-Asp-Glu-Val-Asp-aldehyde, Ac-DEVD-CHO, Promega), 50 μM calpain-2 inhibitor (N-acetyl-leucyl-leucyl-methioninal, calpain-2*i*), 4 nM CaM inhibitor (calmidazolium chloride, CMZ), 20 μM CaMKII inhibitor (KN-93, Calbiochem), 20 μM inactive analogue of KN-93 (KN-92), 20 μM cAMP dependent PKA inhibitor (H-89) for 1 h or 5 mM intracellular calcium chelator {[1,2-bis-(o-aminophenoxy) ethane-N,N,N′,N′-tetraacetic acid tetra (acetoxymethyl) ester], BAPTA/AM)}, 10 mM extracellular calcium chelator [Ethyleneglycol-bis (β-aminoethyl-N,N,N′,N′-tetraacetic acid), EGTA], 10 μM calcium channel blocker nifedipine (Nf), 5 μM calcium channel blocker verapamil (Vp), 100 μM CaMKK inhibitor (STO-609), 20 μM ERK 1/2 inhibitor (U 0126, Calbiochem) for 2 h followed by *A. hydrophila* infection as mentioned above and checked for apoptotic studies and protein assays. The doses of different inhibitors were selected on the basis of inhibitor specificity and cytotoxicity. The HKM treated with the indicated concentrations of the inhibitors remained as viable as control HKM at all time points as determined by the trypan blue (0.4%) dye exclusion method and were maintained during the entire course of the experiment.

### Apoptosis Study

For apoptosis studies the HKM (1×10^6^) were pre-treated separately with or without indicated concentrations of targeted or scrambled siRNAs, indicated concentrations of inhibitors for different time periods then infected with *A. hydrophila* at an MOI of 50 as described above. The HKM were subjected to Hoechst 33342 and Annexin-V-fluorescein isothiocyanate-Propidium Iodide (AV-FITC-PI, BD Pharmingen) staining 24 h p.i. as mentioned earlier [19]. For the Hoechst 33342 study, HKM were collected, washed and fixed with 3.7% paraformaldehyde solution at room temperature. After fixation, the HKM were stained with Hoechst 33342 (2 μg mL^−1^ in 1×PBS) and visualized under fluorescence microscope (×40, Nikon Eclipse 400) within 30 mins of adding the stain. A total of 100 cells were studied in each field and three such fields were included to determine the percentage of apoptotic HKM. For the AV-FITC-PI study the HKM were collected, washed, fixed with 3.7% paraformaldehyde solution and stained with AV-FITC-PI following manufacturer's instructions. The HKM were observed under the fluorescence microscope (×40, Nikon Eclipse 400) within 30 mins of adding the dye. A total of 100 cells were studied in each field and three such fields were included to determine the percentage of apoptotic HKM.

### RNA Isolation, Reverse Transcriptase (RT)-PCR, Cloning, Amplification, Sequencing and Quantitative Real-Time PCR

The HKM (2×10^7^) transfected separately with or without indicated concentrations of targeted or scrambled siRNA were infected with *A. hydrophila* and at indicated time point p.i., culture was terminated, total RNA was isolated using TRIZOL according to manufacturer's instructions. Total RNA dissolved in diethylpyrocarbonate (DEPC) water was treated with deoxyribonuclease I (RNase-free, MBI Fermentas) at 37°C for 30 mins and the DNase was inactivated by incubation with 50 mM EDTA at 65°C for 10 mins. One microgram of total RNA from each sample was reverse transcribed using first strand cDNA synthesis kit as per manufacturer's instructions (MBI Fermentas). The resulting cDNA was then subjected to PCR amplifications using degenerate primers for each gene (Table 1). The amplified product was then gel extracted using QIA quick Gel Extraction Kit (Qiagen) and cloned into pGEM-T EASY vector (Promega) and sequenced (Macrogen). The sequences obtained (Table 2) were aligned to nBLAST and have been submitted to NCBI database.

**Table 1:** List of degenerate primers.

CaM	
Forward Primers	Reverse Primers
FP1 5'- GAR GAR CAR ATY GCW GAR TTC -3'	RP1 5'- YTT CAT YTT YCT DGC CAT CAT -3'
FP2 5'- CAG GAY ATG ATY AAY GAR GT -3'	RP2 5'- CAT CAT YTG KAC RAA YTC TTC -3'

CaMKII*g*	
Forward Primers	Reverse Primers
FP1 5'- GAT CAY CAG AAA CTR GAA CGY -3'	RP1 5'- CTT GTG CTG ATC YTC ATC CCA -3 '
FP2 5'- CAT GAC AGT ATY TCW GAA GAA -3'	RP2 5'- RCA GAC CCA YGG GTG CTT GAG -3 '
FP3 5'- AAY CAY ATY CAC CAG CAT GAC -3'	RP3 5'- CTC AAA GTC YCC ATT GTT -3'

PKACA	
Forward Primers	Reverse Primers
FP1 5'- GAC CAG CAG GGC TAY ATW CAG -3 '	RP1 5'- CTG GTA GAT KGC RAT CCA GTC -3 '
FP2 5'- AAY AAR GCY GTG GAC TGG TGG -3'	RP2 5'- AAA CTC CTT YCC ACA YTT CTC -3'
R = A/G; W = T/A; Y = C/T; K = G/T	

**Table 2:** Gene sequences.

**>CaM (Accession No. KF892532)**
GAGGAGCAAATCGCAGAGTTCAAAGAGGCATTCTCACTCTTTGACAAAGATGGAGATGGCACCATTACGACCAAAGAGTTGGGGACTGTCATGCGCTCTCTTGGCCAGAACCCAACAGAGGCTGAGCTGCAGGACATGATCAATGAAGTGGATGCTGATGGGAATGGGACGATAGACTTCCCAGAGTTCCTGACCATGATGGCAAGGAAGATGAAG
**>CaMKII** ***g*** ** (Accession No. KF892533)**
CATATTCACCAGCATGACATCGTGCACAGAGACCTCAAGCCTGAGAACCTGCTGTTGGCCAGTAAGATGAAAGGAGCAGCAGTTAAGCTGGCTGACTTTGGACTGGCGATTGAGGTACAGGGAGACCAGCAGGCATGGTTTGGCTTTGCAGGCACACCTGGTTACTTATCTCCAGAAGTCCTGAGAAAGGACCCTTATGGCAAACCTGTGGACATTTGGGCTTGTGGAGTTATCCTCTATATTCTTCTTGTGGGATATCCTCCATTCTGGGATGAAGATCAGCATAGGCTCTACCAGCAAATTAAGGCCGGAGCTTATGATTTTCCTTCTCCTGAGTGGGACACCGTTACCCCCGAGGCCAAGAACCTCATCAACCAGATGCTGACCATCAACCCAGCCAAGAGGATCACCGCTGAGCAGGCCCTCAAGCACCCGTGGGTCTG
**>PKACA (Accession No. KF892534)**
CAGCAGGGCTACATACAGGTTACGGATTTCGGCTTTGCCAAGCGTGTAAAGGGCCGGACCTGGACGTTGTGCGGCACTCCTGAGTACCTGGCTCCAGAGATTATCCTCAGTAAAGGCTACAATAAAGCAGTAGATTGGTGGGCTCTGGGTGTGCTGATTTATGAAATGGCTGCAGGATATCCCCCGTTCTTTGCAGACCAGCCCATTCAGATCTACGAGAAGATCGTCTCGGGCAAGGTGCGCTTCCCTTCTCACTTCAGCTCAGACCTGAAGGACCTGCTAAGGAACCTGCTACAGGTAGACCTGACAAAGCGCTTTGGCAACCTCAAGAATGGTGTCAACGACATCAAAGGCCACAAGTGGTTCGCCACCACTGACTGGATTGCCATCTACCAGA

Real-Time PCR for quantitation was done for CaM, CaMKII*g* and PKACA using SYBR green PCR Master Mix (Applied Biosystems) according to the manufacturer's instructions (ABI ViiA, Applied Biosystems) using gene specific primers (CaM forward: 5′-AAGATGGAGATGGCACCATTA-3′ and reverse: 5′-TGGTCAGGAACTCTGGGAAG-3′; CaMKII*g* forward: 5′- TTGTTGACATCTGGGCATGT-3′ and reverse: 5′- CATAAGCTCCGCTTTGATCT-3′; PKACA forward: 5′-AGGTTACGGATTTCGGCTTT-3′ and reverse: 5′-GATCTGAATGGGCTGGTCTG-3′; β-actin forward: 5′- CGAGCAGGAGATGGGAACC-3′ and reverse: 5′-CAACGGAAACGCTCATTGC-3′). The PCR mixture (total volume 10 μl) contained 5 μl of Power SYBR Green, 1 μl of the first-strand cDNA (diluted to 1/100 of the original concentration) and 0.20 μM of forward and reverse primers. Amplification and detection of all genes was performed with ABI ViiA using the following thermal cycling conditions: one cycle of 95°C for 10 mins, 40 cycles of 95°C for 15 s, 60°C for 1 mins. Reactions were performed with cDNAs from six independent experiments and the expression of each transcript was quantified by the comparative ΔΔC_T_ method and normalized to those of β-actin chosen as endogenous control.

### CaM Assay

The cell lysates of HKM (1×10^6^) were pre-treated separately with or without indicated concentrations of CaM-siRNA, scrambled siRNA and inhibitors including BAPTA/AM, EGTA, Vp, Nf for different time periods then infected with *A. hydrophila* at an MOI of 50 as described earlier. The HKM were collected at indicated time point p.i. and checked for CaM concentration by Enzyme Immunoassay Kit (EIA, USCN Life Sciences) as per manufacturer's instruction with brief modifications. Briefly, the lysates along with the standards provided were diluted with diluents, placed at the bottom of each well in triplicate and incubated overnight at 4°C. The liquid of each well was removed carefully and 100 μL of Detection Reagent A was added to each well and incubated for 1 h at 30°C. The solutions were aspirated carefully and each well washed with 200 μL of wash buffer. The Detection Reagent B (100 μL) was added to the wells for 30 mins at 30°C and washed with 200 μL of wash buffer. Ninety microliters of substrate solution was added to each well and the reaction terminated with the addition of 50 μL of stop solution after 25 mins of incubation and the readings taken at 450 nm. The amount of CaM was interpolated from the standard curves obtained by plotting the O.D. of the standards. All chemicals used in the assay were provided with the kit.

### CaMKII*g* Assay

The HKM (1×10^6^) were pre-treated separately with or without indicated concentrations of CaM-siRNA, CaMKII*g*-siRNA, scrambled siRNA and inhibitors including CMZ, KN-93 and KN-92 for different time periods then infected with *A. hydrophila* at an MOI of 50 as described earlier. The HKM were collected at 24 h p.i. and quantitative estimation of CaMKII*g* was done in cell lysates using EIA Kit for CaMKII*g* (USCN) as per manufacturer's instruction. Briefly, HKM were collected by centrifugation, re-suspended in chilled lysis buffer provided with the kit and incubated on ice for 30 mins. Following incubation the cell lysates were collected by centrifugation at 16,000×*g* for 20 mins at 4°C. Hundred microlitres each of standards and cell lysates were added into the wells and incubated for 5 h at 30°C. Following incubation the liquid from each well was removed and 100 μl of Detection Reagent A added to each well and further incubated for 1 h at 30°C. The Detection Reagent A was removed and the wells washed several times with 200 μl of 1× wash solution followed by addition of 100 μl of Detection Reagent B to each well and incubated for 30 mins at 30°C. The wells were washed, 90 μl of substrate added to each well and incubated for 30 mins at room temperature. The reaction was stopped by adding 50 μl stop solution, the readings taken at 450 nm in ELISA plate reader (BMG labtech) and the amount of CaMKII*g* interpolated from the standard curves obtained by plotting the O.D. of the standards. All chemicals used in the assay were provided with the kit.

### cAMP Assay

The cell lysates of HKM (1×10^6^) were pre-treated separately with or without indicated concentrations of inhibitors including KN-93 and KN-92 for different time periods then infected with *A. hydrophila* at an MOI of 50 as described and checked for intracellular cAMP level by ELISA kit (Enzo Life Sciences) as per manufacturer's instruction. Briefly, HKM were collected by centrifugation and resuspended in 100 μl of 0.1 N HCl for 10 mins at room temperature followed by centrifugation at 800×*g* to pellet cellular debris. Hundred microlitres each of standards and supernatant was added to the bottom of appropriate wells, 50 μL each of conjugate and antibody then added and incubated on a plate shaker at room temperature for 4 h. The plate was washed, 200 μL of substrate added to each well and incubated for 1 h at room temperature. The reaction was finally stopped by adding 50 μl of stop solution and reading taken at 405 nm (BMG Labtech). All chemicals used in the assay were provided with the kit.

### Immunofluorescence for PKACA

The HKM (4×10^6^) were pre-treated separately with or without indicated concentrations of CaMKII*g*-siRNA, PKACA-siRNA, scrambled siRNA and inhibitors including KN-93 and H-89 for different time periods then infected with *A. hydrophila* at an MOI of 50 as described earlier. The HKM were collected at 24 h p.i., fixed in methanol/acetic acid (1∶1, v/v) for 30 mins on ice and subsequently incubated with blocking and permeabilizing solution (PBS, 2 mg/ml BSA, 0.2 mg/ml saponin). The cells were washed and incubated with primary antibody for PKACA (1: 200, Sc-903, anti-PKACA raised in rabbit, Santacruz) overnight at 4°C. The HKM were washed, incubated with secondary antibody (1: 250, FITC-conjugated, anti-rabbit, Cell Signalling Technology) for 1 h at room temperature and mounted on microslide with cover slips using fluoroshield. Nuclear staining was done with DAPI (1 μg/ml). The expression and nuclear translocation of PKACA was studied under fluorescence microscope (×100 oil immersion, Zeiss).

### ERK-1/2 Assay

Total ERK was measured using ERK 1/2 EIA (Enzo Life Sciences) and pERK was measured with pThr^202^/Tyr^204^ ERK 1/2 EIA kit (Enzo Life Sciences) [54] using chemicals supplied with the kit. Briefly, HKM (1×10^6^) were pre-treated separately with or without indicated concentrations of PKACA-siRNA, scrambled siRNA and inhibitors including H-89 and U 0126 for different time periods then infected with *A. hydrophila* at an MOI of 50 as described earlier. Total ERK 1/2 and pERK 1/2 activities were measured 24 h p.i. The HKM were collected by centrifugation and re-suspended in chilled lysis buffer and incubated on ice for 30 mins. The cell lysate was collected by centrifugation at 16,000×*g* for 20 mins at 4°C. The assay was performed in a total volume of 100 μl in microtiter plate coated with mouse monoclonal antibody specific to ERK 1/2 provided with the kit. Hundred microliters of the samples were pipetted to the wells and incubated for 2 h at room temperature with shaking. The contents of the wells were washed and 100 μl of polyclonal ERK 1/2 and pERK 1/2 antibody were added separately into respective well and incubated overnight at 4°C. The plates were washed, incubated for 45 mins with HRP-conjugated secondary antibody at room temperature following which 100 μl of TMB substrate containing hydrogen peroxide added to each well and incubated for 30 mins. Finally, 100 μl of stop solution was added and colour development studied at 450 nm. Triplicate sets were prepared containing serially diluted standards, blank (no cell extract), negative control (extract from untreated cells) and *A. hydrophila*-infected HKM. The amounts of ERK 1/2 and pERK 1/2 in the cell lysates were interpolated from the standard curves obtained by plotting the O.D. of the standards. All chemicals used in the assay were provided with the kit.

### Caspase-3 Assay

The caspase-3 (DEVDase) assay was performed using caspase-3 assay kit (Promega) [19]. Briefly, HKM (1×10^6^) were pre-treated separately with or without indicated concentrations of CaM-siRNA, CaMKII*g*-siRNA, PKACA-siRNA, scrambled siRNA and inhibitors including CMZ, KN-93, KN-92, STO-609, H-89 and U 0126 for different time periods then infected with *A. hydrophila* at an MOI of 50 as described earlier. The HKM were collected 24 h p.i. and re-suspended in 50 μL of chilled cell-lysis buffer followed by incubation on ice for 10 mins. The cell lysate were then collected by centrifugation at 10, 000×*g* for 5 mins at 4°C. The caspase-3 (DEVDase) assay was performed in a total volume of 100 μL in 96 well plates. Triplicate wells were prepared containing blank (no cell extract), negative control (extract from untreated cells) and *A. hydrophila*-infected cells. In 10 μL cell extract 32 μL caspase buffer, 2 μL DMSO, 10 μL DTT (100 mM) and 2 μL of the DEVD-pNA substrate were added. The plates were incubated at 37°C for 5 h and absorbance read at 405 nm (BMG Labtech). All chemicals used in the assay were provided with the kit.

### Assay to Detect Intracellular Load of *A. hydrophila*

The PKA specific inhibitor, H-89 was added to HKM (1×10^6^) at different time point *viz*., pre-infection, simultaneous as well as post-infection to *A. hydrophila* infection. In another set of experiment, HKM were transfected with PKACA-siRNA or scrambled siRNA then infected with *A. hydrophila* as described earlier. To quantify the number of intracellular *A. hydrophila*, the HKM were lysed 24 h p.i. with Triton X-100 at a final concentration of 0.1% (v/v) in sterile distilled water. Serial dilutions of lysate from each well were prepared and 0.1 mL of each dilution was plated on nutrient agar and CFU determined after 24 h of incubation at 30°C.

### Statistical Analysis

Data are presented as mean ± SE of number of experiments performed, as indicated in the corresponding figure. Pair-wise comparison was done between group employing paired *t*-test with P<0.05 as the minimum significant level.

## Supporting Information

Figure S1 ***A. hydrophila*** **increases CaM expression in HKM.** HKM were infected with *A. hydrophila* and at indicated time point p.i. CaM expression detected by (A) real time PCR and (B) EIA. Vertical bars represent mean ± SE (n = 6). *P<0.05, compared to HKM. HKM, control head kidney macrophage; HKM+B, HKM infected with *A. hydrophila*.
(TIF)

Figure S2 **Expression of CaMKII** ***g*** **and PKACA at transcript level in** ***A. hydrophila*** **-infected HKM.** HKM were infected with *A. hydrophila* and at indicated time point p.i. (A) CaMKII*g* and (B) PKACA expression detected by real time PCR. Vertical bars represent mean ± SE (n = 6). *P<0.05, compared to HKM. HKM, control head kidney macrophage; HKM+B, HKM infected with *A. hydrophila*.
(TIF)

Figure S3 **Effect of PKA inhibition on intracellular** ***A. hydrophila*** **multiplication.** Intracellular load of *A. hydrophila* was checked in HKM following H-89 addition simultaneous with chloramphenicol and other sets where H-89 was added 15 mins, 30 mins, 60 mins and 120 mins p.i. *A. hydrophila* number was determined by dilution plating on nutrient agar plate. Vertical bars represent mean ± SE (n = 6). **^#^**P<0.05, compared to HKM+B. HKM+B, HKM infected with *A. hydrophila*; HKM+B+H-89 (Simultaneous with Chl), H-89 was added at the time of addition of chloramphenicol; HKM+B+H-89 (15 mins p.i.), H-89 was added 15 mins after the addition of chloramphenicol; HKM+B+H-89 (30 mins p.i.), H-89 was added 30 mins after the addition of chloramphenicol; HKM+B+H-89 (60 mins p.i.), H-89 was added 60 mins after the addition of chloramphenicol; HKM+B+H-89 (120 mins p.i.), H-89 was added 120 mins after the addition of chloramphenicol.
(TIF)
