# Supplementary figures and images for: Role of Calmodulin-Calmodulin Kinase II, cAMP/Protein Kinase A and ERK 1/2 on Aeromonas hydrophila-Induced Apoptosis of Head Kidney Macrophages
Source: PLoS Pathog. 2014 Apr 24;10(4):e1004018. doi: 10.1371/journal.ppat.1004018 (PMC3999153; doi:10.1371/journal.ppat.1004018)

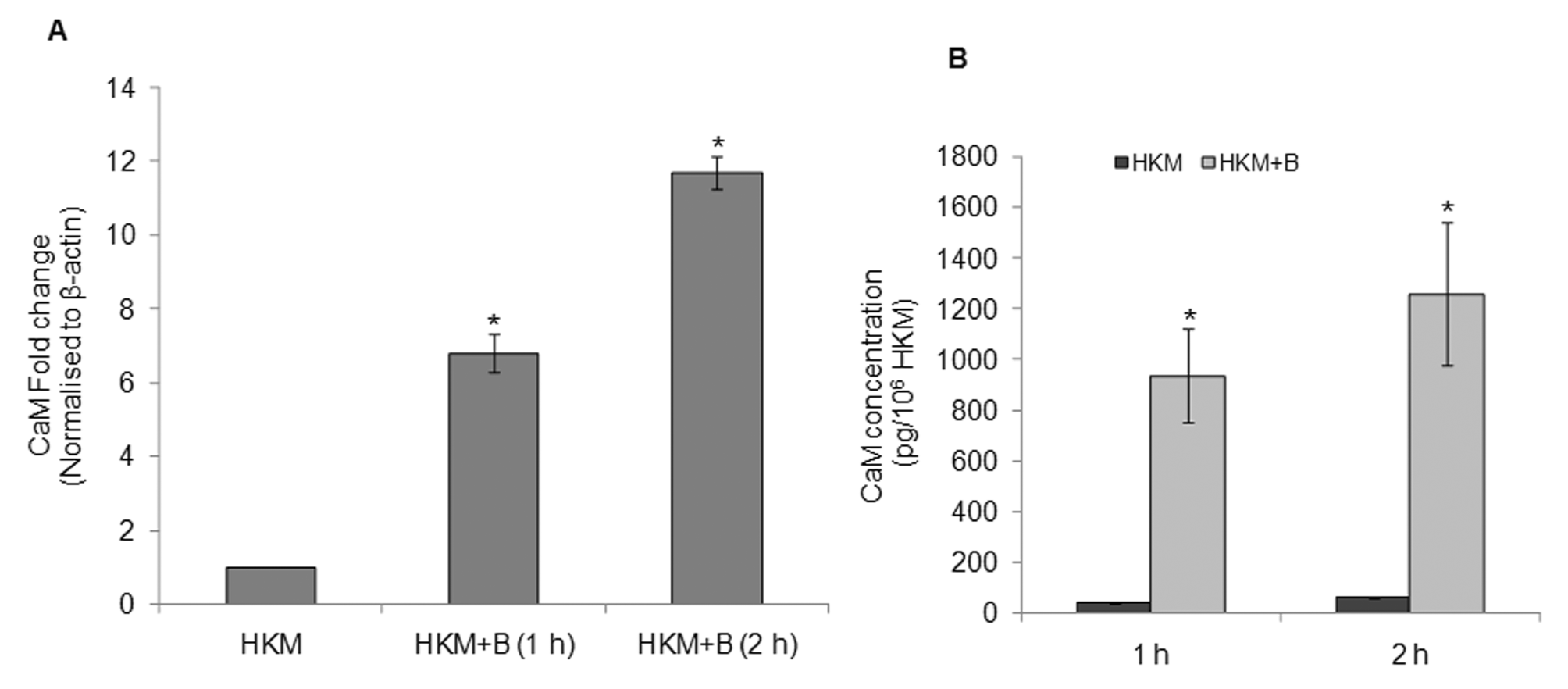

Supplement: Figure S1 — A. hydrophila increases CaM expression in HKM. HKM were infected with A. hydrophila and at indicated time point p.i. CaM expression detected by (A) real time PCR and (B) EIA. Vertical bars represent mean ± SE (n = 6). *P<0.05, compared to HKM. HKM, control head kidney macrophage; HKM+B, HKM infected with A. hydrophila. (TIF) [file ppat.1004018.s001.tif]

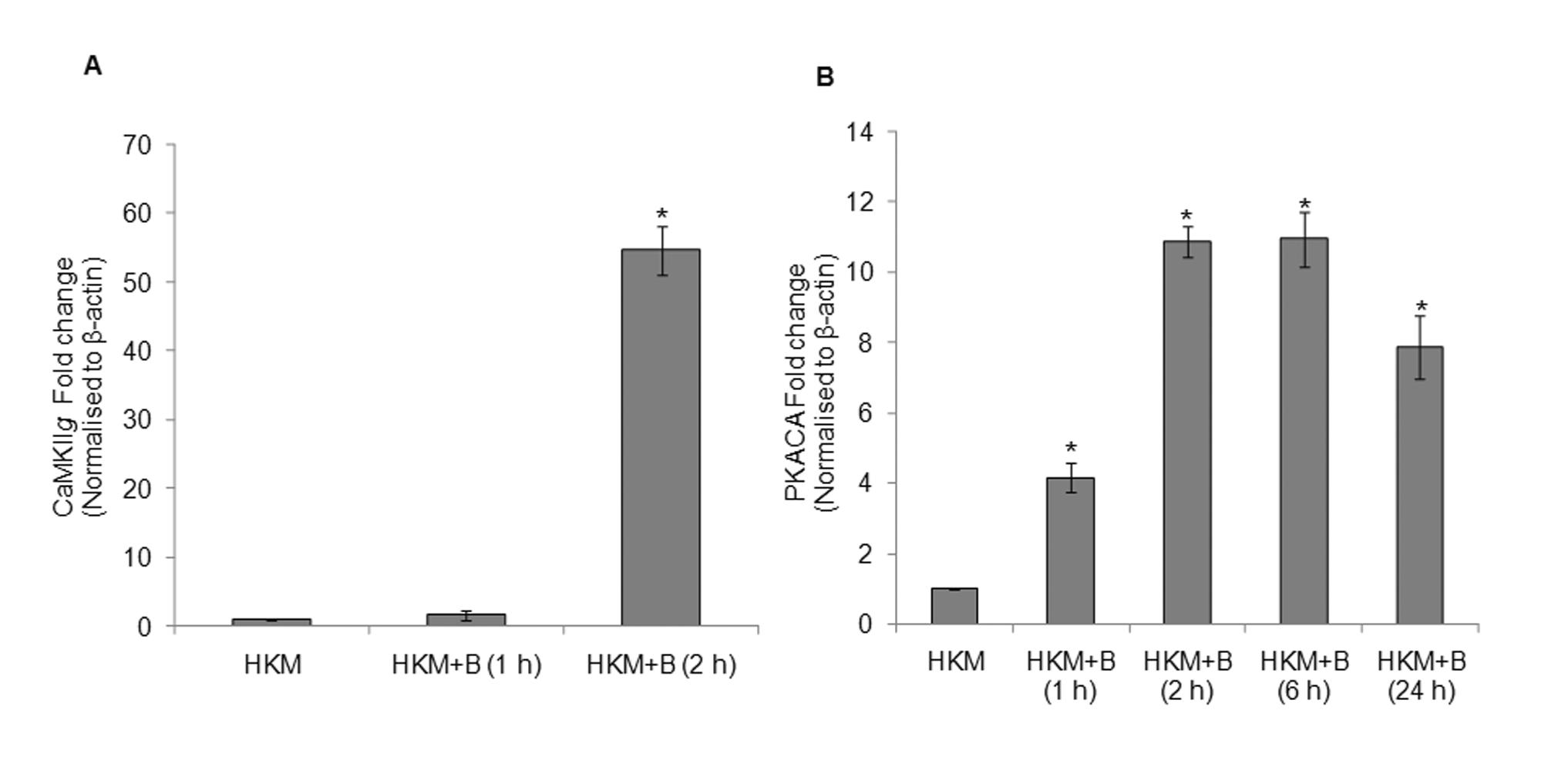

Supplement: Figure S2 — Expression of CaMKII g and PKACA at transcript level in A. hydrophila -infected HKM. HKM were infected with A. hydrophila and at indicated time point p.i. (A) CaMKIIg and (B) PKACA expression detected by real time PCR. Vertical bars represent mean ± SE (n = 6). *P<0.05, compared to HKM. HKM, control head kidney macrophage; HKM+B, HKM infected with A. hydrophila. (TIF) [file ppat.1004018.s002.tif]

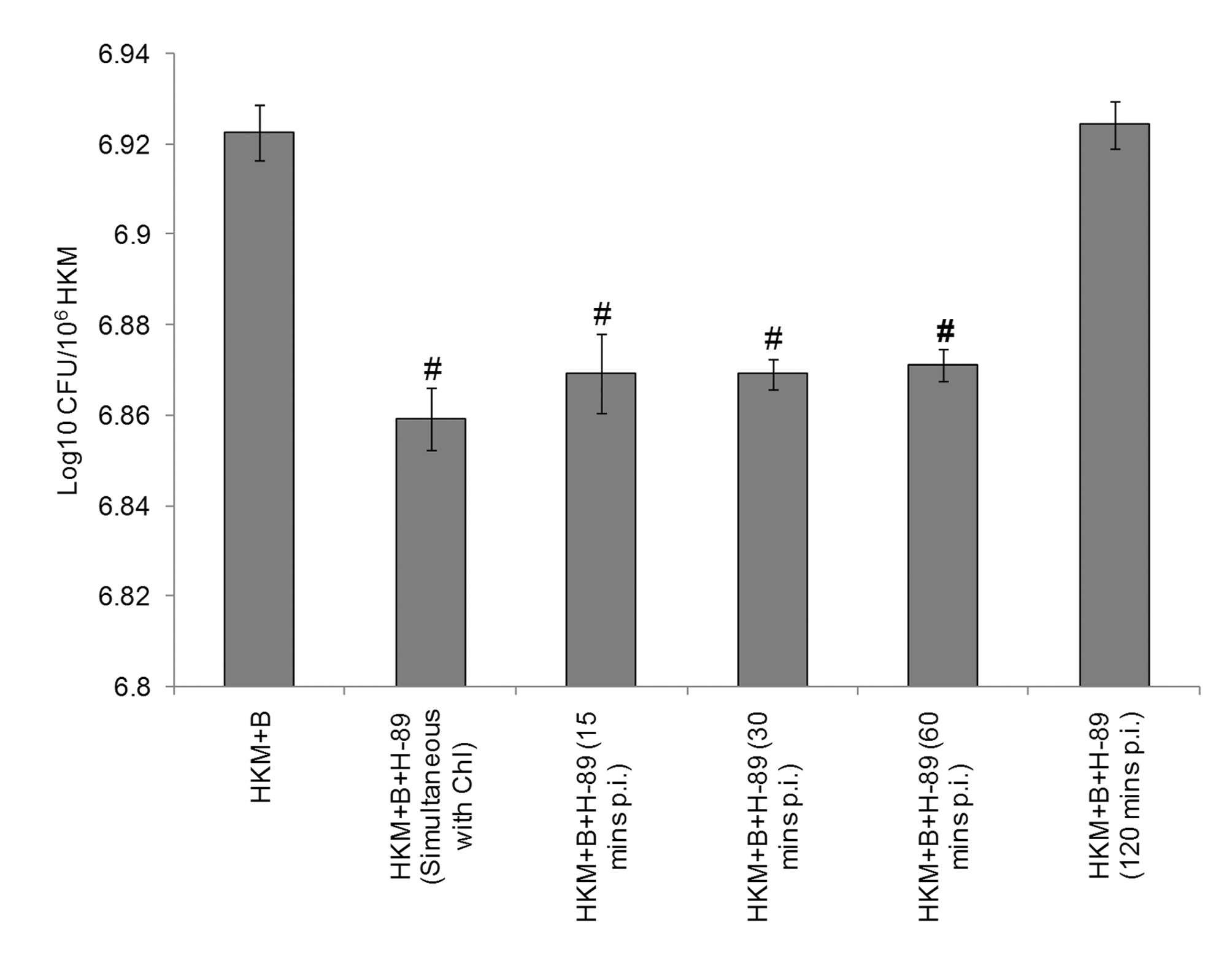

Supplement: Figure S3 — Effect of PKA inhibition on intracellular A. hydrophila multiplication. Intracellular load of A. hydrophila was checked in HKM following H-89 addition simultaneous with chloramphenicol and other sets where H-89 was added 15 mins, 30 mins, 60 mins and 120 mins p.i. A. hydrophila number was determined by dilution plating on nutrient agar plate. Vertical bars represent mean ± SE (n = 6). #P<0.05, compared to HKM+B. HKM+B, HKM infected with A. hydrophila; HKM+B+H-89 (Simultaneous with Chl), H-89 was added at the time of addition of chloramphenicol; HKM+B+H-89 (15 mins p.i.), H-89 was added 15 mins after the addition of chloramphenicol; HKM+B+H-89 (30 mins p.i.), H-89 was added 30 mins after the addition of chloramphenicol; HKM+B+H-89 (60 mins p.i.), H-89 was added 60 mins after the addition of chloramphenicol; HKM+B+H-89 (120 mins p.i.), H-89 was added 120 mins after the addition of chloramphenicol. (TIF) [file ppat.1004018.s003.tif]
